# Neurological manifestations of Erdheim-Chester disease and their management: A scoping review

**DOI:** 10.1097/MD.0000000000041932

**Published:** 2025-03-21

**Authors:** Muhammad Saeed Qazi, Awais Ahmad, Mahreen Mukhtiar, Rizwana Noor, Hassan Ahmed, Shifa Ul Mehmood, Maryam Abbasi, Amna Siddiqui, Muhammad Hasanain, Zain Ul Abideen, Mohammed Mahmmoud Fadelallah Eljack

**Affiliations:** a Medical Research Center, Liaquat University of Medical and Health Sciences, Jamshoro, Pakistan; b Department of Medicine, Saidu Medical College, Swat, Pakistan; c Department of Medicine, Fazai Ruth Pfau Medical College, Karachi, Pakistan; d Department of Medicine, Khyber Medical College, Peshawar, Pakistan; e Department of Medicine, Queen Elizabeth Hospital King’s Lynn, King’s Lynn, United Kingdom; f Department of Medicine, Jinnah Sindh Medical University, Karachi, Pakistan; g Department of Medicine, Karachi Medical and Dental College, Karachi, Pakistan; h Department of Medicine, Dow Medical College, Karachi, Pakistan; i Department of Medicine, Quaid-e-Azam Medical College, Bahawalpur, Pakistan; j Community Department, University of Bakht Alruda, Ad Duwaym, Sudan.

**Keywords:** Erdheim-Chester disease, management, neurological manifestation, scoping review

## Abstract

**Background:**

Erdheim–Chester disease (ECD) is characterized by the excessive production and accumulation of histiocytes, particularly foamy histiocytes encircled by fibrosis, in various organs. ECD can affect a person’s long bones, skin, lungs, brain, heart, and other tissues and organs.

**Methods:**

In February 2024, we conducted a thorough literature search on main databases to find studies published between 2021 and 2024. We used keywords like “ECD CNS,” “ECD Neuro,” “Erdheim Chester Disease CNS,” “histiocytosis,” and “Erdheim Chester Disease Neurology.” We first produced 461 articles and then limited the selection process to 29 articles using an English language filter and an abstract analysis. In the end 16 articles were added in the review on ECD with neurological symptoms.

**Results:**

Ataxia, cranial neuropathies, and cognitive impairment were the main clinical findings. Histopathology showed that 88% of the cases had histiocyte infiltrates with CD68 + histiocytes, 50% had a BRAF mutation, and 83.3% had an expanding mass. The most common treatment was vemurafenib, followed by steroids, surgery, and chemotherapy. Results revealed 15.78% mortality, 26.31% progression, and 31.57% improvement or stabilization. In most cases, steroid medication proved useless; however, vemurafenib and radiation therapy helped some patients’ symptoms.

**Conclusion:**

An early diagnosis of the disease is important for timely intervention, to prevent fatal outcomes. Vemurafenib was the only drug which proved its efficacy against the disease, as compared to other drugs. This study presents valuable insights for the neurology of ECD and highlights the benefits of a comprehensive management for this complex condition.

## 1. Introduction

Erdheim–Chester disease (ECD) is an exceedingly rare non-Langerhans cell histiocytosis (NLCHs) first identified in 1930 by Jakob Erdheim and William Chester. To date, about 1500 cases have been reported, the incidence of which appears to be increasing, likely due to increased recognition of the disease. This condition, initially termed “lipoid granulomatosis,” was considered a nonneoplastic inflammatory condition but has now been reclassified as hematopoietic malignancy by the World Health Organization and categorized within the “L” (Langerhans) group of the revised histiocytosis classification by the Histiocyte Society in 2016.^[1,2]^

ECD is characterized by the infiltration of tissues by histiocytes, specifically foamy histiocytes surrounded by fibrosis, setting it apart from other histiocytic disorders such as Langerhans cell histiocytosis (LCH).^[3]^ Clinical presentations of ECD vary widely, ranging from asymptomatic bone lesions to life-threatening multisystemic involvement. Skeletal manifestations, notably osteosclerosis in the long bones, are common, often leading to bone pain and pathological fractures. Neurological involvement is a prominent feature, with manifestations including cerebellar and pyramidal syndromes, seizures, headaches, central diabetes insipidus, exophthalmos and cognitive impairment. In one systematic review, 56% of the patients were reported to have central nervous system (CNS) involvement in ECD.^[4]^ Importantly, CNS involvement is associated with significant morbidity and mortality in ECD patients.^[5]^

Diagnostic criteria for ECD typically involve a combination of imaging studies, tissue biopsies, and immunohistochemical analysis. Characteristic histopathological findings include the presence of CD68-positive and CD1a-negative histiocytes, along with fibrosis in affected tissues.^[6]^ Recent advances in molecular biology have elucidated mutations in the mitogen-activated protein kinase pathway, particularly BRAF, in a majority of ECD cases.^[2]^ This discovery has led to the development of targeted therapeutic options such as vemurafenib, offering hope for improved management strategies.

Despite these advancements, ECD remains a diagnostic and therapeutic challenge due to its rarity and complexity. The heterogeneous nature of the disease, coupled with its multifaceted clinical manifestations, often leads to delays in diagnosis and suboptimal management.

Our scoping review aims to provide a comprehensive summary of neurological involvement in ECD, associated clinical, biochemical, and radiological findings, potential treatment options, patient outcomes, and demographic correlations.

## 2. Methods

Arksey and O Malloy scoping review methodology was employed to guide our study.^[7]^ Ethical approval was not required for the conduct of this review.

### 2.1. Research question

We selected the research question “What are the clinical manifestations, radiological findings, diagnostic approaches, treatment modalities, management strategies and outcomes in ECD patients with central nervous system involvement?”

### 2.2. Literature search approach

As this is an update of a previous systematic review, we will only consider articles dated from July 15, 2021 onwards. The results of this updated search will be combined with eligible case reports and case studies identified in the prior review, which will serve as an important source for prior case reports and case studies. In March 2024, we carried out a comprehensive literature search on main databases to identify articles published between July 15, 2021 and March 15, 2024. Our search strategy included the following keywords “ECD CNS,” “ECD Neuro,” “Erdheim Chester Disease CNS,”” histiocytosis” and “Erdheim Chester Disease Neurology.” Boolean operators (OR, AND, NOT) were employed to combine the keywords and associated terms during the literature search.

### 2.3. Inclusion and exclusion criteria

Consistent with the previous systematic review, we included case reports and case series of ECD patients presenting with neurological symptoms. Because English is the worldwide scientific language, only articles written in English were selected. Studies describing cases of ECD with non-neurological manifestations, articles written in a language other than English, and articles republishing previously reported cases were excluded.

### 2.4. Abstract and full paper review

Our initial search strategy using the publication dates we selected (July 15, 2021–February 17, 2024) yielded 264 results. English language filter was applied, resulting in the exclusion of 18 articles. The process of choosing the articles was carried out in 2 stages. In the first stage, 3 members individually reviewed the titles and abstracts of the 246 articles based on the inclusion criteria. This process aimed to ascertain whether their content potentially addresses the review questions. Of 246 articles, 50 were excluded after abstract review, leaving 196 articles. The full texts of the 196 selected articles were examined in the second stage. Like the first stage, 3 authors independently assessed the full text of the selected articles to determine if they met the objectives of the review. Group discussions were held among the researchers to ensure consistency in the review process and resolve any discrepancies regarding the inclusion or exclusion of the articles. Ultimately, 16 articles met the eligibility criteria to be included in our review.

### 2.5. Data extraction

We extracted the following data from each selected article: first author name, publication year, age at diagnosis, gender, CNS imaging location, clinical and radiological findings, treatment received, management strategies, follow-up duration and outcome. Specifically, we recorded the frequency of main clinical findings, diagnostic features, histopathological findings, treatment modalities used, management approaches, morbidity, and mortality data such as frequency of disease progression, improvement, and death. The lead author extracted all the relevant information, which was subsequently verified by the last author and supervisor of this project.

### 2.6. Data synthesis and analysis

We used Google spreadsheet to enter the extracted data as this makes it easier to organize and analyze the data. Categorical variables such as gender, clinical symptoms, treatment modalities and outcome were presented using descriptive statistics – frequencies and percentages. Continuous variables, for instance, age at diagnosis, follow-up duration was interpreted by measures of central tendency (mean or median) and dispersion (standard deviation or range). A *P*-value of <.05 was considered statistically significant.

### 2.7. Tabulations

To present the findings, we sorted the data into tables according to various characteristics, each focusing on a distinct aspect of ECD in individuals with central nervous system involvement. These tables were organized around essential categories such as demographics, clinical features, diagnostic features, treatments, outcomes, CNS imaging locations, and management strategies. This approach contributed to a deeper comprehension of the disease and its management.

## 3. Results

### 3.1. Comprehensive literature search

**Figure 1.**
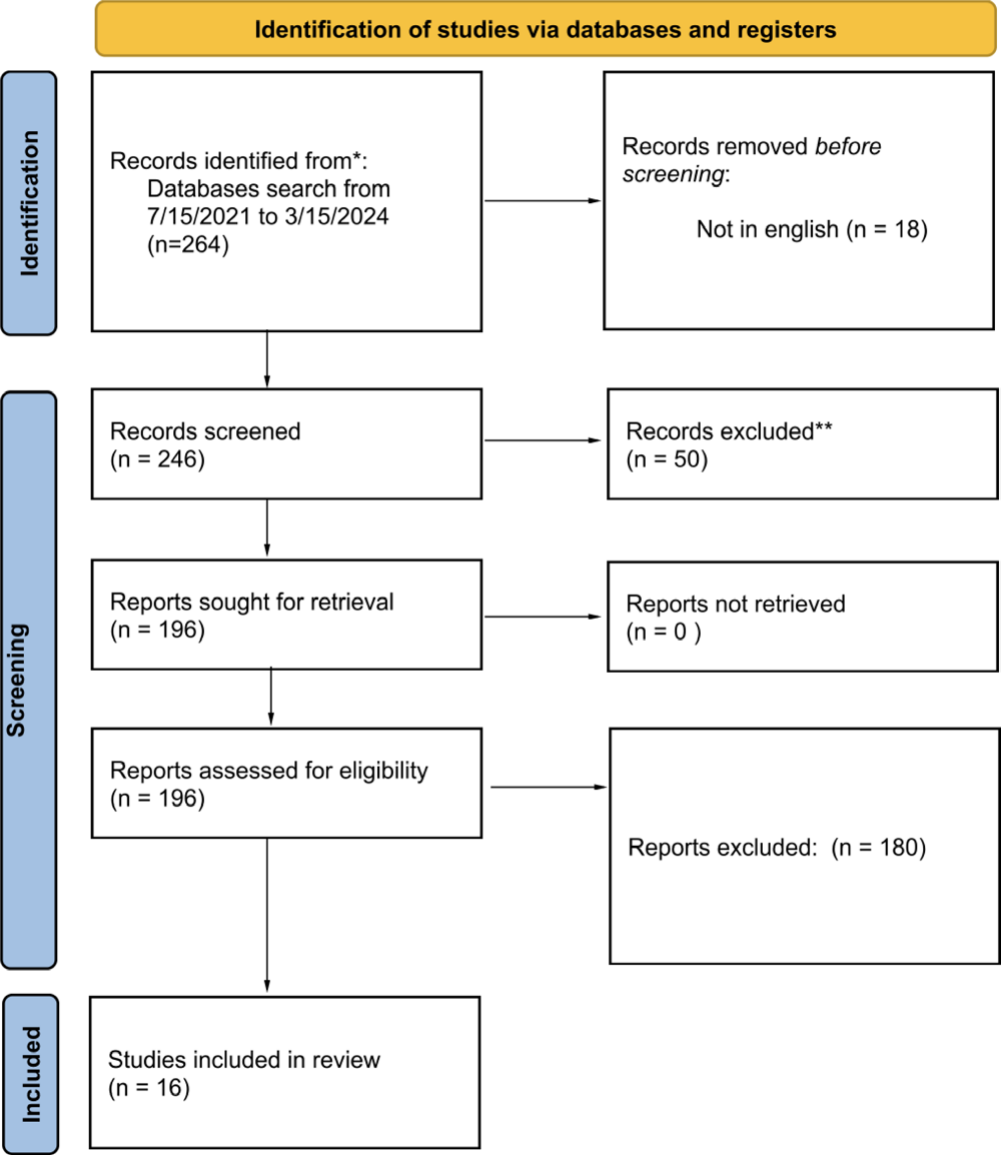
Flow diagram of the selection process of the records.

The selection procedure of all records received through the literature search is illustrated in Fig. 1. A total of 264 records were screened. Of these, 18 records were removed after being found duplicated. Two hundred forty-six articles were screened for title and abstract and 50 articles were removed for not matching criteria. One hundred ninety-six articles were assessed for eligibility, out of which 180 articles were removed due to various reasons. Finally, 16 studies are included in this review. Detailed search strategy utilized for each database is mentioned in Table S1, Supplemental Digital Content, http://links.lww.com/MD/O535.

### 3.2. General study characteristics

The study design incorporated case reports and case series of ECD patients presenting with neurological symptoms, extracted between 2021 and 2024. Additionally, systematic reviews from studies conducted before 2021 were included to gain insights into previous findings and trends. Within this framework, these studies focused on detailing clinical and radiological findings, treatment modalities, and outcomes. The study involved a significant demographic analysis of patients. The cohort comprised an equal distribution of male (n = 28, 50.00%) and female (n = 28, 50.00%) participants. The age of the participants spanned from 2.5 to 82 years, with a mean age of onset at 51.25 years (±21.84). The follow-up duration for these patients ranged from 1 to 120 months.

Table 1 summarizes the demographic information of patients with neurological symptoms included in the scoping review and publication years ranging from 2014 to 2024. Detailed characteristics are mentioned in Table S2, Supplemental Digital Content, http://links.lww.com/MD/O537.

**Table 1:** Baseline characteristics of patients with Erdheim-Chester disease and neurological manifestations.

Characteristics	
Male (n)	28 (50%)
Female (n)	28 (50%)
Age (years; mean ± SD)	41.08 ± 23.0
Follow up duration (months; mean ± SD)	60.5 ± 34.6
Age of onset (years; mean ± SD)	51.3 ± 21.8

### 3.3. Clinical findings and diagnostic features of ECD

The neurological presentations of the review exhibited a diverse array of symptoms. Cranial neuropathies were observed in 6 individuals, accounting for 50.5% of cases. Vomiting was reported by 2 patients, making up 11.1% of the cohort. Neck pain was experienced by 3 patients, representing 16.6%. Headaches were noted in 7 cases, corresponding to 38.8%. Ataxia was the most frequent symptom, affecting 13 individuals, or 72% of the participants. Cognitive impairment was significant, reported in 10 cases, which is 55.5% of the cohort. Pyramidal symptoms were rare, with only 1 case (5.5%). Limb weakness was prevalent in 11 patients, accounting for 61% of the cohort. Hydrocephalus and focal motor seizures were each observed in 2 individuals, both representing 11.1%. Vision loss or vision symptoms were noted in 8 cases (44%), while cerebellar syndrome was present in 4 cases (22.2%). Dysarthria was seen in 8 patients (44.4%), and dysphagia was reported in 2 cases (11.1%). Hemorrhage, urinary incontinence, and syncope/loss of consciousness were each recorded in 1 case (5.55%). Dizziness was reported by 6 patients, accounting for 33.3%. Paresthesia hypesthesia, and scanning speech were each noted in 3 individuals (16.6%). Lastly, aphasia was observed in 1 case, representing 5.55% of the cohort.

In this study, the pattern of neurological involvement and associated histopathological findings were thoroughly evaluated. Among the cohort, 15 individuals (83.3%) presented with an expansile mass. Additionally, BRAF mutation was identified in 9 cases, accounting for 50% of the participants. Histopathological analysis revealed that histiocyte infiltrates were present in 16 cases, representing 88% of the cohort. Similarly, CD68 + histiocytes were detected in 16 cases (88%). Main clinical and diagnostic features of patients are summarized in Table 2.

**Table 2:** Neurological presentations and histopathological features in Erdheim-Chester disease patients.

Neurological presentation	Frequency (n)	Frequency (%)
Cranial neuropathies	6	50.5
Vomiting	2	11.1
Neck pain	3	16.6
Headache	7	38.8
Ataxia	13	72.0
Cognitive impairment	10	55.5
Pyramidal	1	5.5
Limb weakness	11	61.0
Hydrocephalus	2	11.1
Vision loss/vision symptoms	8	44.0
Cerebellar syndrome	4	22.2
Dysarthria	8	44.4
Dysphagia	2	11.1
Hemorrhage	1	5.6
Urinary incontinence	1	5.6
Dizziness	6	33.3
Syncope/loss of consciousness	1	5.6
Focal motor seizure	2	11.1
Paresthesia/hypesthesia	2	11.1
Scanning speech	3	16.6
Aphasia	1	5.6
*Neurological involvement pattern*		
Expansile mass	15	83.3
*BRAF mutation*	9	50.0
*Histopathological findings*		
Histiocytes infiltrates	16	88.0
CD68+	16	88.0

This combination of neurological and histopathological data provides a comprehensive overview of the disease characteristics, summarizing the main clinical findings and diagnostic features of ECD patients presenting with neurological symptoms.

### 3.4. Treatment

The cohort received a variety of treatments targeting their neurological conditions. Steroids were administered to 4 patients, representing 21.05% of the group. Surgical intervention or debulking was performed in 3 cases (15.7%), while chemotherapy was also utilized in 3 patients (15.7%). Vemurafenib was the most frequently used treatment, given to 5 patients, accounting for 26.31%. Radiation therapy was administered to 1 patient (5.26%), and cobimetinib was also used in 1 case (5.26%). Additionally, various other treatments were employed, each in a single patient (5.26%), including levetiracetam, sulfadiazine, lamotrigine, folinic acid, a combination of temozolomide and autologous stem cell transplant, pyrimethamine, interferon, cladribine, cyclophosphamide, ceftriaxone, amphotericin, ampicillin, clofarabine, vancomycin, and methotrexate. Treatment is summarized in Table 3.

**Table 3:** Treatment and clinical outcomes in Erdheim-Chester disease patients with neurological involvement.

Treatment	Number	Percentage
Steroids	4	21.05%
Surgery/debulking	3	15.7%
Chemotherapy	3	15.7%
Vemurafenib	5	26.31%
Radiation	1	5.26%
Cobimetinib	1	5.26%
Levetiracetam	1	5.26%
Sulfadiazine	1	5.26%
Lamotrigine	1	5.26%
Folinic acid	1	5.26%
Temozolomide, autologous stem cells transplant	1	5.26%
Pyrimethamine,	1	5.26%
Interferon	2	10.52%
Cladribine	3	15.78%
Cyclophosphomide	1	5.26%
Ceftriaxone	1	5.26%
Amphotericin	1	5.26%
Ampicillin	1	5.26%
Clofarabine	1	5.26%
Vancomycin	1	5.26%
Methotrexate	1	5.26%
Outcome	Number	Percentage
Improvement or stabilization of symptoms	6	31.57%
Progression	5	26.31%
Death	3	15.78%
No improvement with steroids	4	21.05%
Improvement of symptoms by vemurafenib	2	10.52%
Improvement of symptoms by radiotherapy	1	5.26%
Death not secondary to CNS disease	1	5.26%
Improvement of symptom by interferon alpha	1	5.26%
Improvement of symptoms by Vemurafenib	1	5.26%
Improvement of symptoms by debulking	1	5.26%
Improvement in symptoms by cobimetinib	1	5.26%

### 3.5. Outcomes

The outcomes of the treatments were diverse. Improvement or stabilization of symptoms was observed in 6 patients, which corresponds to 31.57% of the cohort. However, 5 patients (26.31%) experienced progression of their condition, and 3 patients (15.78%) unfortunately passed away. Notably, 4 patients (21.05%) did not show improvement with steroid treatment. Vemurafenib led to symptom improvement in 2 patients (10.52%), while radiotherapy resulted in symptom improvement in 1 patient (5.26%). Additionally, there was a reported case of death that was not secondary to CNS disease. Improvement of symptoms was also noted in 1 patient each (5.26%) following treatments with interferon-alpha, vemurafenib (again noted separately), debulking surgery, and cobimetinib. Summary of outcomes reported is given in Table 3.

## 4. Discussion

ECD is an exceedingly rare NLCHs with around 1500 cases reported since it was first diagnosed in 1930. Our qualitative review comprises a thorough analysis of case reports and case series, initially presenting with signs and symptoms of the central nervous system. Our study primarily focused on the CNS presentations of ECD. However, it is important to note that nearly half of the patients also exhibit extra-CNS manifestations, which significantly impact the prognosis of the disease (see Table 4). The skeletal system is the most commonly affected area, followed by cardiovascular, endocrine, cutaneous, urological, nephrological, and pulmonary involvement.^[8]^

**Table 4:** Clinical features of Erdheim-Chester disease: CNS versus extra-CNS involvement.

Clinical feature of patients	CNS involvement	Extra-CNS involvement
Fatigue	Chronic, significant	Common in systemic diseases like ECD
Fever	Present, often as part of systemic symptoms	Present, often without other systemic symptoms
Thirst & polyuria	Due to central diabetes insipidus	Not commonly reported
Leg pain	Related to bone involvement (sclerotic lesions)	Common, related to skeletal involvement
Xanthoma of upper eyelids	May or may not appear	Common as a cutaneous manifestation
Disturbance of consciousness	Present, related to CNS involvement	Not typically reported
Headaches or visual disturbances	Present, related to CNS involvement	Not typically reported
Skin manifestations	Present as yellowish-brown plaques/nodules	Present as maculopapular lesions (e.g., on trunk and extremities)
Bone involvement	Symmetrical sclerotic lesions in long bones	Symmetrical sclerotic lesions in long bones
Neurological deficits	Present, such as dizziness, ataxia, dysarthria	Not typically present
Cardiac symptoms (pericardial effusion, heart failure)	May occur in severe cases	May occur but not as commonly as in CNS-involved cases
Pulmonary symptoms	Respiratory distress due to lung involvement	Pulmonary symptoms such as pleural effusion may occur
Abdominal pain	May occur with CNS involvement	May occur, especially related to peritoneal or renal involvement
Imaging findings (MRI/CT/PET)	Shows infiltration in brain and spinal cord	Bone marrow involvement and organ infiltration such as kidneys, pericardium, etc.
Skeletal involvement	Symmetrical lesions in femurs, tibias	Symmetrical lesions in femurs, tibias, ribs, vertebrae
Renal involvement	Not typically seen	Renal infiltration leading to hydronephrosis, fibrosis
Hematological abnormalities	May show anemia, thrombocytopenia	May show thrombocytosis, anemia
Endocrine symptoms	Diabetes insipidus	Less common but may occur in advanced stages
Other systemic symptoms (e.g., fatigue, malaise, weight loss)	Common due to systemic involvement	Common in systemic diseases like ECD

Consistent with our findings, the study by Zenggang Pan and Bette K. Kleinschmidt-DeMasters reported that less than one-third of cases initially presented with CNS involvement, while 50% of cases eventually developed CNS association.^[9]^ ECD presented with multiple CNS symptoms like ataxia (72%), limb weakness (61%), cranial neuropathies (50.50%), cognitive impairment (55.50%), vision loss/visual problems (44%), Dysarthria (44.40%), headache (38.80%) and many more like loss of consciousness, paresthesia/hypesthesia, dysphagia, vomiting, hydrocephalus and many others (Table 2). These findings are consistent with the study conducted by Louisa C. Boyd et al in 2020, which reported extensive central nervous system involvement. Key clinical findings included cognitive impairment (52%), cerebellar ataxia (46%), cranial neuropathies (61%), peripheral neuropathies (56%), and upper motor neuron involvement (30%). Additionally, coordination errors were observed in 33% of patients, while 26% exhibited an inability to perform rapid alternating movements. Oculomotor abnormalities were present in 44% of the cohort.^[10]^

CNS lesions in the form of expansile masses were seen in 83% of the patients (Table 2). According to the study conducted by Florence Lachenal et al in 2005, 3 patterns of lesions were observed i.e. Infiltrative pattern (44%), meningeal patterns (37%), and composite one comprising both meningeal and infiltrative pattern (19%).^[11]^ Around 88% of the cases were positive for CD68 + histiocytes and infiltration (Table 2) which is similar to the study of EL diamond et al stating that ECD histiocytes were positive for CD68, CD 163, Factor XIIIa, S100 (occasionally) and negative for CD1a and CD207.^[6]^ BRAF mutation was found in 50% of the cases (Table 2) similar to Gaurav Goyal et al which states that BRAF V600E mutation was frequent and seen in 50% of the population.^[12]^

However, differentiating ECD from other NLCH disorders with CNS involvement, such as Juvenile Xanthogranuloma and Rosai-Dorfman Disease (RDD), is essential as each has distinct clinical, radiological, morphological and histopathological, immunophenotypic features. CNS involvement occurs in approximately 40% of patients, with clinical and radiologic signs present in as many as 90% of cases. Predilection sites include the posterior fossa brain parenchyma and spinal cord, but the pituitary, dura, facial sinuses, and orbits can also be affected. Patients frequently experience cognitive impairment, ataxia, peripheral neuropathy, and headaches. Skeletal involvement, particularly bilateral and symmetric sclerotic lesions of the long bones, is a hallmark of ECD.^[1,2]^

In contrast, LCH predominantly involves the hypothalamic-pituitary axis particularly, pituitary stalk, pineal gland, and circumventricular regions, with T2 hyperintense, gadolinium-enhancing lesions.^[3]^ Parenchymal involvement is rare but may present as symmetrical signal changes in the cerebellar gray matter, pons, basal ganglia, and globus pallidum. LCH lesions are generally smaller and more localized compared to those in ECD.^[4]^ RDD rarely involves the CNS. When present, it predominantly affects the dura, often mimicking meningiomas on imaging, with parenchymal infiltration being rare.^[5]^ Histiocytic sarcoma (HS), a malignant disorder, can involve the CNS as part of systemic disease or as a primary manifestation. It presents enhanced mass lesions on imaging that mimic glioblastoma or metastases. This disorder is highly aggressive, with a poor prognosis due to its rapid progression and limited treatment options.^[6]^

Histopathologically, ECD is characterized by foamy histiocytes within a bland inflammatory xanthogranulomatous inflammation, often with fibrosis.^[7]^ LCH, in contrast, shows abnormal dendritic cells with CD1a, Langerin, and S100 protein positivity.^[3]^ RDD is identified by enlarged histiocytes with abundant pale cytoplasm and the hallmark feature of emperipolesis (the engulfment of lymphocytes or plasma cells by histiocytes).^[5]^ HS is distinguished by pleomorphic histiocytes and is typically positive for CD68, CD163, CD4, and lysozyme, with frequent mutations in the mitogen-activated protein kinase/ERK pathway.^[6]^ Immunophenotypically, ECD histiocytes express CD163 and strongly express factor XIIIa, while lacking CD1a, langerin, and ZBTB46, which are markers of LCH.^[7,9]^ ECD is also negative for S100, fascin, and OCT2, further differentiating it from RDD ^[9]^. HS shows CD68, CD163, CD4, and lysozyme expression.

Multiple treatment modalities were used for patients presenting with neurological manifestations of ECD. These included steroids (21.05%), debulking surgeries (15.7%), chemotherapy (15.7%), radiations (5.26%), and vemurafenib (26.31%) which inhibits the mutant BRAF V600E kinase and is the most frequently used treatment modality. Many other options were also used among which interferons and cladribine are worth mentioning (Table 3). The same treatment options were used in ECD: a systematic review by Cives M, et al published in 2015.^[4]^ The most effective treatment was vemurafenib, while IFN alpha showed promising results. The outcomes of the different treatment modalities are shown in Table 3. According to the study by Haroche J et al 2013, vemurafenib is the most effective treatment for patients with ECD BRAF V600E mutation positive while IFN alpha shows a subtle and slow effect on the disease activity.^[13]^ The prognosis of the patients with ECD depends on the system involved and when presented with neurological manifestations it has made the prognosis very poor. With 3 of the patients representing 15.7 % of the sample size unfortunately passed away (Table 3). With the advancement in the field of genetics and molecular biology, the presentations of ECD have become chronic, not a fatal illness, that is, the overall survival rate has increased from 43% (1996) to 83% (2018).^[12]^

The findings of this scoping review on ECD with neurological presentation show significance in the diagnosis, treatment, and prognosis. The clinicians must consider ECD in their differential diagnosis if patients present with atypical CNS symptoms accompanied by bone pain and multisystem involvement. While managing such patients this review enhances the diagnostic accuracy and targeted therapies for the treatment and good prognosis of patients. Keeping in mind the 2 important factors of poor prognosis, that is, ECD with CNS involvement and no proper diagnosis, we recommend that the researchers delve into the molecular mechanism of ECD to uncover new agents for the targeted therapy of the disease. Conduct longitudinal studies to investigate these therapeutic agents’ efficacy and safety. A proper diagnostic criterion should be searched for new genetic and molecular biomarkers to diagnose ECD.

The review provides a detailed overview of case reports that are solely presented with CNS symptoms and fills the gap in the existing literature which usually does not focus only on the CNS presentation of ECD. The review results are consistent with the existing literature and generalizable across different patient populations. The review provides deep insights into the clinical manifestations, diagnosis, histopathology, other treatment modalities, and how to improve the outcomes of these modalities. The review promotes a multidimensional approach and application of advanced genetics and molecular biology in treating ECD. This systematic review includes only 16 case reports. It does not include articles published in languages other than English, potentially resulting in omitting crucial data, which might limit the generalizability of the findings.

However, the review has some limitations, such as it does not include extensive epidemiological data apart from basics like gender and age, due to the exceedingly rare nature of the disease. Thus, there is a paucity of data comparing epidemiological data in CNS-involved versus noninvolved patients, with only 1500 known cases worldwide, and likely not all reported with a complete set of epidemiology, limited our review to build a solid epidemiological picture of ECD with current studies. Further, due to scarce literature available in terms of RCTs and observational studies, we refrained from conducting a meta-analysis of case series which is relatively a weak study design that would not generalize the findings.

## 5. Conclusion

This scoping review summarizes the clinical features, radiological appearances, therapeutic strategies, and outcomes of ECD patients with neurological symptoms. Based on the included studies cranial neuropathies, ataxia, limb weaknesses, and cognitive impairment were frequently reported symptoms. An early recognition of this disease is important for timely interventions, to prevent mortality. Vemurafenib, a BRAF inhibitor, was found to ease the symptoms while other drugs showed no significant impact. One potential multi-dimensional approach to this is to investigate combining vemurafenib with MEK inhibitors or immunotherapies to augment efficacy. The genotype of the malignant cells may also affect the treatment modalities that the patient receives. Studies suggest that techniques such as immunohistochemistry may have promise as a way to optimize therapy. In addition, the course of the disease and death also leaves a lot of room for research needed to develop more potent therapies.

This study provides novel findings for the neurology of ECD and highlights the benefits of multidisciplinary management for a complex disease like ECD.

## Author contributions

**Conceptualization:** Muhammad Saeed Qazi.

**Data curation:** Muhammad Saeed Qazi, Mahreen Mukhtiar, Maryam Abbasi.

**Formal analysis:** Muhammad Saeed Qazi, Awais Ahmad, Mahreen Mukhtiar, Maryam Abbasi.

**Investigation:** Muhammad Saeed Qazi, Amna Siddiqui.

**Methodology:** Mahreen Mukhtiar, Rizwana Noor, Shifa Ul Mehmood, Maryam Abbasi.

**Project administration:** Awais Ahmad, Mahreen Mukhtiar, Shifa Ul Mehmood.

**Resources:** Rizwana Noor, Hassan Ahmed.

**Supervision:** Amna Siddiqui, Muhammad Hasanain, Zain Ul Abideen.

**Validation:** Shifa Ul Mehmood, Zain Ul Abideen.

**Visualization:** Rizwana Noor, Mohammed Mahmmoud Fadelallah Eljack.

**Writing – original draft:** Muhammad Saeed Qazi, Awais Ahmad, Mahreen Mukhtiar, Hassan Ahmed, Shifa Ul Mehmood, Maryam Abbasi.

**Writing – review & editing:** Muhammad Saeed Qazi, Rizwana Noor, Amna Siddiqui, Muhammad Hasanain, Zain Ul Abideen, Mohammed Mahmmoud Fadelallah Eljack.

## Supplementary Material

SUPPLEMENTARY MATERIAL
