# Supplementary material for: Neurological manifestations of Erdheim-Chester disease and their management: A scoping review
Source: Medicine (Baltimore). 2025 Mar 21;104(12):e41932. doi: 10.1097/MD.0000000000041932 (PMC11936638; doi:10.1097/MD.0000000000041932)
Supplement: SUPPLEMENTARY MATERIAL [file medi-104-e41932-s001.docx]

| **Databases** | **Search Strings** |
| --- | --- |
| **PubMed** | (("erdheim chester disease"[MeSH Terms] OR ("erdheim chester"[All Fields] AND "disease"[All Fields]) OR "erdheim chester disease"[All Fields] OR ("erdheim"[All Fields] AND "chester"[All Fields] AND "disease"[All Fields]) OR "erdheim chester disease"[All Fields] OR "ECD"[All Fields] OR ("histiocytosis"[MeSH Terms] OR "histiocytosis"[All Fields] OR "histiocytoses"[All Fields])) AND ("ieee conf commun netw secur"[Journal] OR "clin nurse spec"[Journal] OR "clin nurs stud"[Journal] OR "cns"[All Fields] OR ("neurology"[MeSH Terms] OR "neurology"[All Fields] OR "neuro"[All Fields] OR "neuros"[All Fields]) OR ("neurology"[MeSH Terms] OR "neurology"[All Fields] OR "neurology s"[All Fields]) OR ("central nervous system"[MeSH Terms] OR ("central"[All Fields] AND "nervous"[All Fields] AND "system"[All Fields]) OR "central nervous system"[All Fields]))). |
| MEDLINE | "erdheim chester disease"[MeSH Terms] OR ("erdheim chester"[All Fields] AND "disease"[All Fields]) OR "erdheim chester disease"[All Fields] OR ("erdheim"[All Fields] AND "chester"[All Fields] AND "disease"[All Fields]) OR "erdheim chester disease"[All Fields] |
| CENTRAL | "erdheim chester disease"[MeSH Terms] OR ("erdheim chester"[All Fields] AND "disease"[All Fields]) OR "erdheim chester disease"[All Fields] OR ("erdheim"[All Fields] AND "chester"[All Fields] AND "disease"[All Fields]) OR "erdheim chester disease"[All Fields] |
| Google Scholar | "erdheim chester disease"[MeSH Terms] OR ("erdheim chester"[All Fields] AND "disease"[All Fields]) OR "erdheim chester disease"[All Fields] OR ("erdheim"[All Fields] AND "chester"[All Fields] AND "disease"[All Fields]) OR "erdheim chester disease"[All Fields] |
| Embase | "erdheim chester disease"[MeSH Terms] OR ("erdheim chester"[All Fields] AND "disease"[All Fields]) OR "erdheim chester disease"[All Fields] OR ("erdheim"[All Fields] AND "chester"[All Fields] AND "disease"[All Fields]) OR "erdheim chester disease"[All Fields] |

**Supplementary Table 1:** Search Strategies Used in Different Databases
