# Supplementary material for: Neurological manifestations of Erdheim-Chester disease and their management: A scoping review
Source: Medicine (Baltimore). 2025 Mar 21;104(12):e41932. doi: 10.1097/MD.0000000000041932 (PMC11936638; doi:10.1097/MD.0000000000041932)
Supplement: SUPPLEMENTARY MATERIAL [file medi-104-e41932-s002.docx]

| **First Author, Year** | **Country** | **Study Design** | **Association** | **Comments** | **Text Availabilty** | **Diagnostic Findings**[**[1]**](https://docs.google.com/document/d/1XgyTZGdIfIlUN-iifecRayhBL1aeImjN45FYi-iZstk/edit#heading=h.1fob9te) | **Treatment/Surgical Intervention** | **Unique Finding**[**[2]**](https://docs.google.com/document/d/1XgyTZGdIfIlUN-iifecRayhBL1aeImjN45FYi-iZstk/edit#heading=h.3znysh7) | **BRAF** |
| --- | --- | --- | --- | --- | --- | --- | --- | --- | --- |
| Suheyla et al 2021 | Turkey | case report | yes | In a child with refractory, multisystem histiocytosis and atypical presentations, mixed LCH/ECD should be suspected in the differential diagnosis. | full text needed |  |  |  |  |
| Y J Yang et al 2021 | China | case report |  | * | full text needed |  |  |  |  |
| Nuh et al 2021 | Turkey | case report | yes | Neurologic involvement is presented with fewer than 50% of the patients, and diabetes insipidus and ataxia syndromes are the most common presentations. The importance of FDG PET/CT for assessing the extent of skeletal disease, deciding the site for biopsy, and evaluating treatment response is well known.8–10 In this case, we demonstrated the role of FDG PET/CT in the differential diagnosis and the extent of central nervous system involvement in a patient presenting with neurological symptoms | full text needed |  |  |  |  |
| Sydney et al 2021 |  | case report | yes | ECD patients may undergo multiple evaluations anThe diagnosis of ECD is challenging mainly because of the insidious onset, diverse organ systems affected and non-specific morphological or immunophenotypic characteristics of histiocytes on histopathologyd workups, which may delay diagnosis and treatment due to the variation in presentation and the wide variety of organ systems affected. | available | MRI ,biopsy to confirm xanthomatous histiocytes and to test for BRAF and MAPK-ERK pathway mutations.a full-body Positron Emission Tomography-Computed Tomography (PET-CT) scan or a Computed Tomography (CT) with a contrast of chest, abdomen and pelvis. | Most patients with ECD require treatment except for asymptomatic and single organ ECD. Steroids, radiation, and surgery may be utilized as an adjunct therapy to help relieve symptoms.Patients with positive BRAF-V600 mutations, cardiac/neurologic disease, or end-organ failure may receive vemurafenib, dabrafenib, or encorafenib as first-line therapy; MEK inhibitors may be considered otherwise.  Neurosurgery performed a suboccipital craniotomy, partially resected the cerebellar mass, and placed a parietal to frontal shunt catheter | It was recently recognized as a neoplastic disorder after identifying an activating mutation of the MAPK pathway |  |
| Rahul et al 2022 |  | case report | yes | In summary, due to its rarity, diagnosis of ECD, a rare non-Langerhans cell histiocytosis, is difficult even when it classically presents with symmetric, lower extremity osteosclerosis, multi-organ involvement, and histopathology documenting invasion by Langerin-negative histiocytes with surrounding fibrosis. CNS-only disease is extremely rare, and particularly tricky to diagnose and manage. | available | Computed tomography (CT) imaging of the chest, abdomen, and pelvis was unremarkable. However, magnetic resonance imaging (MRI) with contrast of the brain revealed diffuse, enhancing masses including most prominently a 2.4 cm × 1.4 cm lesion centered in the dorsal medulla | Thus, our experience suggests that in appropriately selected cases, palliative CSI may safely offer improved local control and symptomatic relief for CNS disease. | case presentation: medulla complicated by respiratory failure and motor deficits, which represents, to our knowledge, the first patient with disseminated leptomeningeal disease (LMD) at diagnosis without systemic disease. |  |
| Jerome et al 2022 | Finlad | case report | yes | However, this case report highlights that ECD should be screened in patients with unexplained delirium, as treatment with interferon followed by BRAF inhibitor improves psychiatric features and induces partial remission in neurodegenerative ECD. | available | brain MRI showing hyperintense signal in the pons on T2-FLAIR sequence/ axial CT scan showing sheathing of aorta adventitia/ axial CT scan showing bilateral perinephric fat infiltration defined as “hairy-kidney”/ Whole-body CT scan (18FDG-PET) showed peri-nephric fat infiltration and aorta adventitia sheathing with radiotracer uptake in the pons, vessels, peri-nephric fat, and bone lesions, which was characteristic of ECD | Treatment with interferon resulted in the resolution of delirium, and treatment with BRAF inhibitor subsequently resulted in a partial remission of all active sites | This case highlights that delirium can be the first manifestation of neurodegenerative ECD. |  |
| Rajesh et al 2022 |  | case report | yes | This case report highlights the importance of considering ECD in differential diagnoses when a patient presents with CNS symptoms of unclear etiology. Additionally, this emphasizes the importance of biopsy and IHC testing of cellular pathology to determine the correct course of treatment. Furthermore, this paper reinforces data found in Diamond et al.’s study that cobimetinib effectively prevents ECD disease progression. | available | An MRI showed an increase in parafalcine mass size and chronic thrombosis of the superior sagittal sinus. The mass was debulked and biopsied. The IHC panel of this tissue was positive for Factor XIIIa, suggesting ECD, however negative for BRAF. | The fact that our patient is BRAF negative may explain why she did not show a response to interferon but did to cobimetinib which is a particularly effective therapy for BRAF negative ECD variants. | This patient has several atypical features including an initial presentation with atraumatic subdural hematoma, concomitant meningioma on the last biopsy, poor response to interferon, need for serial debulking, excellent response to cobimetinib, and lack of any other organ involvement over the last almost eight years. |  |
| Tomoya et al 2022 |  | case report | yes | Thus, distinguishing between ECD with CNS involvement and TE may be difficult using only clinical and radiologic findings, and the diagnosis should be made by taking into account the histopathologic findings, involvement of other organs, and responses to anti-Toxoplasma treatment. | available | Contrast-enhanced CT of the trunk revealed soft-tissue shadows in areas surrounding the descending thoracic aorta and left kidney |  | This case suggests the difficulty of distinguishing ECD with CNS involvement from toxoplasmic encephalitis and the possibility of a relationship between the pathogeneses of ECD and infection with T. gondii |  |
| Daniela et al 2022 |  | case report | yes | In summary, despite ECD being a rare neurological disease, neurologists must consider this entity as a putative cause of the cerebellar syndrome with dentate nuclei involvement in cerebral MRI, which is potentially treatable | available | The brain MRI presented T2 hyperintense lesions involving the dorsal part of the pons, lower midbrain tegmentum, middle cerebellar peduncles, and bilateral dentate nuclei, without gado- linium enhancement (CT) was performed showing an increase in soft tissue density surrounding both kidneys (corresponding to typical “hairy kidneys”) and at the aortoiliac bifurcation, suggesting a possible periaortitis | in our patient sev- eral analyses were done in bone marrow tissue to exclude BRAF mutations. The negative results of these tests led to the choice of IFN-α therapy. |  |  |
| Christopher et al 2022 |  | case report | yes | Our case of hypoglycorrhachia from a neoplasm, ECD, is a rare presentation of a rare diagnosis, a true challenge for the diagnostician. ECD may present as meningitis though. | available |  | Response to treatment has been improved with kinase inhibitor therapies targeting the mitogen-activating protein kinase pathway | . ECD may present as meningitis though, ECD can present with meningitis with hypoglycorrhachia. | Immunohistochemical stains for CD1a and BRAF were negative.The negative CD1a  stain essentially ruled out a Langerhans histiocytosis. Diagnosis  was made of ECD. |
| Vittorio et al 2023 | Italy | literature review | yes | that the characteristic brain MRI findings in “pure “neurological ECD patients consists of a diffuse, exclusive, or prominent involvement of the infratentorial compartment mainly involving the cerebellum, the brainstem, and the cerebellar peduncles, usually without contrast enhancement/ Another diagnostic clue for neurological ECD might be represented by the presence of signs of sinusitis and/or mastoiditis on the brain MRI/ A definite diagnosis of ECD, according to current diagnostic criteria for ECD requiring histology confirmation, was reached in three patients of our cohort: in particular in Pt 2, one nodular lesion configured a histiocytic sarcoma the presence of bulbar affect in about 30% of cases, supporting that this symptom might represent a “red flag” to suspect neurological ECD when combined with ataxia and the presence of peculiar infratentorial neuroimaging lesions. | available | Brain MRI showed T2/FLAIR hyperintense signal alterations without contrast enhancement variably involving the white matter of cerebellar hemispheres, middle cerebellar peduncles, dentate nuclei, pons, and midbrain and cerebral peduncles in all cases |  | This case series illustrates the occurrence of Erdheim–Chester disease presenting exclusively with neurological manifestations. |  |
| Shreyashi et al 2023 |  | case report | yes | To conclude, CNS involvement in ECD needs to be detected early because it is associated with a poor prognosis. Isolated CNS involvement with posterior fossa involvement and obstructive hydrocephalus presenting in the third decade is unusual, and a high index of suspicion is required to diagnose and treat early to avoid the usual dismal outcomes./ Diabetes insipidus (DI) and bone pains that predate neurological symptoms often direct the physician to the diagnosis of ECD/ Orbital masses are majorly seen with ECD | available | Magnetic resonance imaging (MRI) brain showed T2 hyperintense lesions in the cerebellum and pons with intense contrast enhancement and obstructive hydrocephalus |  |  | These cells were also positive for BRAFV600E by immunohistochemistry, (IHC) indicating that these cells were non-Langerhans cell types and favored the diagnosis of non-histiocytic disorder compatible with ECD |
| Charlotte et al 2023 | Paris | cross sectional | yes | ECD was associated with a great lifetime prevalence of depressive and anxiety disorders. This association was not explained by the depressogenic effects of treatments. Regarding the relationship between mood and inflammation, it could be assumed that the high level of depression would be related to inflammatory processes in ECD.ECD is also associated with changes in personality and in neuropsychological performances | available |  |  | ECD patients exhibited high level of past depressive disorder (80%) and anxiety disorder, especially agoraphobia (29%). They revealed personality changes, especially with high agreeableness (t = 3.18, p < 0.005) and high conscientiousness (t = 3.81, p < 0.001). Neuropsychological assessments showed impairments in attention (GZ: t = 16.12, p < 0.0001, KL: t = 37.01, p < 0.0001) and episodic memory performances (STIR: t = − 3.01, p = 0.006, LTFR: t = − 2.87, p = 0.008, LTIR: t = − 3.63, p = 0.001). Executive functions, such as flexibility, inhibitory control, were unimpaired |  |
| Sophie et al 2023 |  | case report |  | * | full text needed |  |  |  |  |
| J C Benson et al 2023 |  |  |  | * | full text needed |  |  |  |  |
| Samantha et al 2023 |  | review article |  | * | full text needed |  |  |  |  |
| Elke et al 2023 |  | case report |  | * | full text needed |  |  |  |  |
| Amir et al 2023 |  | case report |  | * | full text needed |  |  |  |  |
| Koramadai et al 2023 |  | case report | yes | ECD can manifest initially with neurological symptoms such as stroke, meningitis, and brain lesions and with vision symptoms and retro-orbital masses. Skeletal manifestation is more common, where bone scan is very useful and will show characteristic finding in ECD. FDG PET/CT shows that brain and orbital lesions can occur in malignancy. The coated aorta, which is a characteristic finding in ECD, if seen in those cases, we should raise the possibility of ECD. | available | Fluorine-18 fluorodeoxyglucose positron emission tomography/computed tomography (F-18 FDG PET/CT) plays a vital role in the diagnosis, detection of disease extent and severity, assessment for an appropriate biopsy, and treatment response.the image findings of FDG PET/CT in a case of brain and orbital lesions, which showed hypermetabolic coated aorta which is a specific clue to the diagnosis of ECD. |  | Exophthalmos (retro-orbital space infiltration), “coated aorta” (circumferential soft-tissue sheathing of the thoracic aorta), and “hairy kidney” (soft-tissue rind of perirenal infiltration) are typical manifestations in patients with ECD |  |
| Caren et al 2023 |  | case report | yes | In this case, PET/CT imaging identified both CNS and long bone lesions, even in the absence of bone pain, and retrospectively correlated the histopathologic diagnosis of ECD with prior imaging findings./ It is important to note the progression of differential diagnoses in this case alongside the serial imaging findings, with many conclusions considered in retrospect. | available | Imaging findings demonstrated retroperitoneal fibrosis and long bone osteosclerosis with increased fluorodeoxyglucose uptake that, together with the neuropathologic findings, were diagnostic of ECD. |  | This case of biopsy-proven ECD is unique in that the singular symptom was seizures well controlled with medical management in the presence of similarly located bilateral anterior mesial temporal lobe lesions. Although ECD is rare intracranially, its variable imaging presentation, including the potential to mimic seizure-associated medial temporal lobe tumors, emphasizes the need for a wide differential diagnosis. | A non–Langerhans cell histiocytosis with a BRAF V600E mutation was identified on pathology. |
| Giuseppe et al 2023 |  | case report | yes | ECD, an uncommon histiocytic neoplasm, presents diverse clinical manifestations, posing significant diagnostic challenges, particularly due to its rarity. In our case, the absence of systemic symptoms led to a diagnostic focus on neurological manifestations, supported by MRI findings. | available |  |  | . In our case, the patient presented solely with neurological signs/symptoms, in the absrnce of systemic symptoms making the diagnosis challenging |  |
| Valentina et al 2024 |  |  |  | * | full text needed |  |  |  |  |
| Carlen et al 2024 |  |  |  | * | full text needed |  |  |  |  |
| Merve et al 2024 |  | case report |  | * | full text needed |  |  |  |  |
| Zehra et al 2024 |  | case report |  | * | full text needed |  |  |  |  |

**Supplementary Table 2:** Summary of studies on Erdheim-Chester Disease (ECD) with Neurological Involvement
